# Neutral impact of mTOR inhibitors on cardiovascular outcomes after kidney transplantation

**DOI:** 10.1093/ckj/sfag101

**Published:** 2026-03-25

**Authors:** Eulàlia Domingo-Pinent, Judit Cacho, Matthias Cassia, Verónica Torres, Hugo Vergara, Aileen Herceda, Paula Juarez, Ángela Gonzalez-Rojas, Diana Rodríguez-Espinosa, Enrique Montagud-Marrahi, Carolt Arana, Alicia Molina-Andujar, Pedro Ventura-Aguiar, Nuria Esforzado, Ignacio Revuelta, José Vicente Torregrosa, Frederic Oppenheimer, Antonio Francino, Fritz Diekmann, David Cucchiari

**Affiliations:** Unidad de Trasplante Renal (UTR), Instituto Clínico de Nefrología y Urología (ICNU), Hospital Clínic, Barcelona, Spain; Unidad de Trasplante Renal (UTR), Instituto Clínico de Nefrología y Urología (ICNU), Hospital Clínic, Barcelona, Spain; Unidad de Trasplante Renal (UTR), Instituto Clínico de Nefrología y Urología (ICNU), Hospital Clínic, Barcelona, Spain; Department of Nephrology, Hospital Universitario Rio Ortega, Valladolid, Spain; Department of Nephrology, Hospital General Universitario de Castellón, Castellón, Spain; Unidad de Trasplante Renal (UTR), Instituto Clínico de Nefrología y Urología (ICNU), Hospital Clínic, Barcelona, Spain; Unidad de Trasplante Renal (UTR), Instituto Clínico de Nefrología y Urología (ICNU), Hospital Clínic, Barcelona, Spain; Unidad de Trasplante Renal (UTR), Instituto Clínico de Nefrología y Urología (ICNU), Hospital Clínic, Barcelona, Spain; Unidad de Trasplante Renal (UTR), Instituto Clínico de Nefrología y Urología (ICNU), Hospital Clínic, Barcelona, Spain; Unidad de Trasplante Renal (UTR), Instituto Clínico de Nefrología y Urología (ICNU), Hospital Clínic, Barcelona, Spain; Unidad de Trasplante Renal (UTR), Instituto Clínico de Nefrología y Urología (ICNU), Hospital Clínic, Barcelona, Spain; Unidad de Trasplante Renal (UTR), Instituto Clínico de Nefrología y Urología (ICNU), Hospital Clínic, Barcelona, Spain; Unidad de Trasplante Renal (UTR), Instituto Clínico de Nefrología y Urología (ICNU), Hospital Clínic, Barcelona, Spain; Laboratori Experimental de Nefrologia I Trasplantament (LENIT), Institut d’Investigacions Biomèdiques August Pi i Sunyer (IDIBAPS), Barcelona, Spain; Unidad de Trasplante Renal (UTR), Instituto Clínico de Nefrología y Urología (ICNU), Hospital Clínic, Barcelona, Spain; Unidad de Trasplante Renal (UTR), Instituto Clínico de Nefrología y Urología (ICNU), Hospital Clínic, Barcelona, Spain; Laboratori Experimental de Nefrologia I Trasplantament (LENIT), Institut d’Investigacions Biomèdiques August Pi i Sunyer (IDIBAPS), Barcelona, Spain; Unidad de Trasplante Renal (UTR), Instituto Clínico de Nefrología y Urología (ICNU), Hospital Clínic, Barcelona, Spain; Unidad de Trasplante Renal (UTR), Instituto Clínico de Nefrología y Urología (ICNU), Hospital Clínic, Barcelona, Spain; Instituto Clínico Cardiovascular, Hospital Clínic, Barcelona, Spain; Unidad de Trasplante Renal (UTR), Instituto Clínico de Nefrología y Urología (ICNU), Hospital Clínic, Barcelona, Spain; Laboratori Experimental de Nefrologia I Trasplantament (LENIT), Institut d’Investigacions Biomèdiques August Pi i Sunyer (IDIBAPS), Barcelona, Spain; Red de Investigación Renal (REDINREN), Madrid, Spain; Unidad de Trasplante Renal (UTR), Instituto Clínico de Nefrología y Urología (ICNU), Hospital Clínic, Barcelona, Spain; Laboratori Experimental de Nefrologia I Trasplantament (LENIT), Institut d’Investigacions Biomèdiques August Pi i Sunyer (IDIBAPS), Barcelona, Spain; Red de Investigación Renal (REDINREN), Madrid, Spain; Universitat de Barcelona, Barcelona, Spain

**Keywords:** acute myocardial infarction, cardiovascular outcomes, kidney transplantation, MACE, mTOR inhibitors

## Abstract

**Background:**

In kidney transplantation, it remains unclear whether mammalian target of rapamycin inhibitors (mTORi) improve cardiovascular outcomes—through plaque stabilization and attenuation of left ventricular remodeling—or worsen them by deranging glucose and lipid metabolism. We aimed to assess the true impact of this drug class on major cardiovascular outcomes in a deeply phenotyped cohort with long-term follow-up.

**Materials and methods:**

All patients transplanted at our center between 1st June 2013 and 31st December 2019 (*n* = 845) were screened for inclusion. A total of 492 patients were selected through 1:1 propensity score matching (PSM) based on 18 key donor and recipient variables. All patients received tacrolimus (TAC), steroids, and either mTORi (*n* = 246) or mycophenolic acid (MPA) (*n* = 246). The primary outcome was major adverse cardiovascular events (MACE), defined as non-fatal myocardial infarction, non-fatal stroke, or cardiovascular death.

**Results:**

Baseline variables were adequately balanced after PSM (absolute standardized difference <0.10). Over a mean follow-up of 4.81 ± 2.49 years, MACE occurred in 78 patients (15.9%), with no significant difference between the mTORi and MPA groups (HR[95% CI] 0.84[0.54–1.30], *P* = 0.443). Subgroup analyses—including patients with diabetes, prior MACE, stable immunosuppression, or pre-transplant ischemia testing—also showed no differences. Independent predictors of MACE were age (HR[95% CI] for upper tertile 2.17[1.38–3.42], *P* < 0.001), dialysis vintage (HR[95% CI] for upper tertile 1.91[1.22–3.01], *P* = 0.005), prior myocardial infarction (HR[95% CI] 2.12[1.19–3.78], *P* = 0.011), and deceased vs. living donor graft (HR[95% CI] 3.42[1.47–7.92], *P* = 0.004).

**Conclusions:**

Cardiovascular disease after kidney transplantation occurs due to non-modifiable risk factors and does not appear to be related to baseline immunosuppression.

KEY LEARNING POINTS **What was known:**

- Cardiovascular disease remains the leading cause of death after kidney transplantation, with recipients facing a twofold higher risk than the general population despite improved survival compared to dialysis patients.
- Standard immunosuppressive regimens, including CNIs and corticosteroids, contribute to metabolic disturbances that may sustain this excess cardiovascular risk.
- Mammalian target of rapamycin (mTOR) inhibitors offer potential cardiovascular benefits but also cause dyslipidemia and hyperglycemia; current evidence on their net cardiovascular impact—especially when used de novo with CNIs—is limited and largely registry-based.

**This study adds:**

- To address this unanswered clinical question, we analyzed data from a deeply phenotyped cohort of kidney transplant recipients (initial population, *n* = 845) with exhaustive analysis of pre-transplant cardiovascular work-up and risk factors. Primary outcome was the incidence of major adverse cardiovascular events (MACEs) over a long-term follow-up of 4.81 ± 2.49 years.
- Patients taking tacrolimus and mTOR inhibitors (TAC + mTORi) were propensity-score matched 1:1 to patients taking TAC and mycophenolate (TAC + MPA) according to 18 key donor and recipient factors, obtaining optimal balance (final *n* = 492). Importantly, no differences were noted between the two groups for risk factors, previous cardiovascular events and pre-transplant work-up.
- No differences were noted in MACEs after transplantation between the two patients’ groups. Sensitivity analyses in patients with diabetes, previously known cardiovascular disease, stable immunosuppression, or pre-transplant ischemia testing also showed no differences. Independent predictors of MACE were non-modificable risk factors, including age, dialysis vintage, prior infarction, and deceased donor.

**Potential impact:**

- These findings suggest that the choice of immunosuppression is unlikely to influence the incidence of MACEs after kidney transplantation, supporting clinicians in prioritizing other clinical considerations (e.g. immunologic risk, side-effect profile) when choosing immunosuppressive regimens.
- Since MACE risk appears mainly driven by non-modifiable factors (e.g. age, dialysis vintage, and prior infarction), strategies to prevent cardiovascular events should shift from drug selection toward targeted risk stratification and aggressive management of traditional risk factors.
- Policy and practice may benefit from integrating standardized cardiovascular screening and long-term risk reduction programs into transplant pathways, rather than modifying baseline immunosuppression for presumed cardiovascular benefit.

## INTRODUCTION

Cardiovascular disease (CVD) is the leading cause of death after kidney transplantation (KT), accounting for 20%–35% of overall mortality [1, 2]. Compared to the age-matched general population, kidney transplant recipients face a twofold higher risk of CVD [3]. Although dialysis patients bear an even higher cardiovascular burden, up to 10–20 times greater [4], KT fails to fully normalize this risk. This residual risk likely stems from a combination of pre-existing conditions and transplant-related factors. Among the latter, immunosuppressive medications may play a role in sustaining elevated cardiovascular risk post-transplant. Calcineurin inhibitors (CNIs) and corticosteroids—core components of most immunosuppressive protocols—are known to negatively affect glucose and lipid metabolism. These agents may provoke or intensify metabolic disturbances, including diabetes, elevated cholesterol and triglyceride levels [5]. Additionally, CNIs are associated with hypertension in a dose-dependent manner [4, 5]. Mycophenolate (MPA), which often completes the classical immunosuppressive triad, is considered metabolically neutral and does not significantly influence cardiovascular risk. An alternative to MPA is the use of mammalian target of rapamycin inhibitors (mTORi), such as sirolimus and everolimus. These drugs have demonstrated equivalence to the traditional CNI + MPA regimen in preventing rejection and maintaining graft function and survival [6]. However, like CNIs and steroids, mTORi can also disrupt metabolic balance by increasing glucose and lipid levels, potentially contributing to the development and progression of atherosclerosis [7]. On the other hand, mTORi may offer some cardiovascular benefits, including atheroma plaque stabilization, attenuation of left ventricular remodeling, and enabling reductions in CNI exposure [8–10]. Although mTORi have shown non-inferiority to standard immunosuppressive regimens regarding graft survival and rejection, their effect on post-transplant cardiovascular risk remains uncertain. Existing evidence is largely limited to registry-based studies lacking detailed clinical phenotyping or in which mTORi were not consistently used in combination with CNI [2, 11]. Given their complex profile—with both potentially harmful and protective cardiovascular effects—there is a need for better evidence regarding their impact when used de novo in combination with CNIs. To address this gap, we analyzed a deeply phenotyped cohort of kidney transplant recipients. Patients initiating a *de novo* regimen of mTORi alongside the CNI tacrolimus (TAC) were propensity-score matched 1:1 with recipients receiving the conventional TAC and MPA regimen. Our primary aim was to compare the long-term incidence of major adverse cardiovascular events (MACE) between the two treatment groups.

## MATERIALS AND METHODS

### Setting

**Figure 1:**
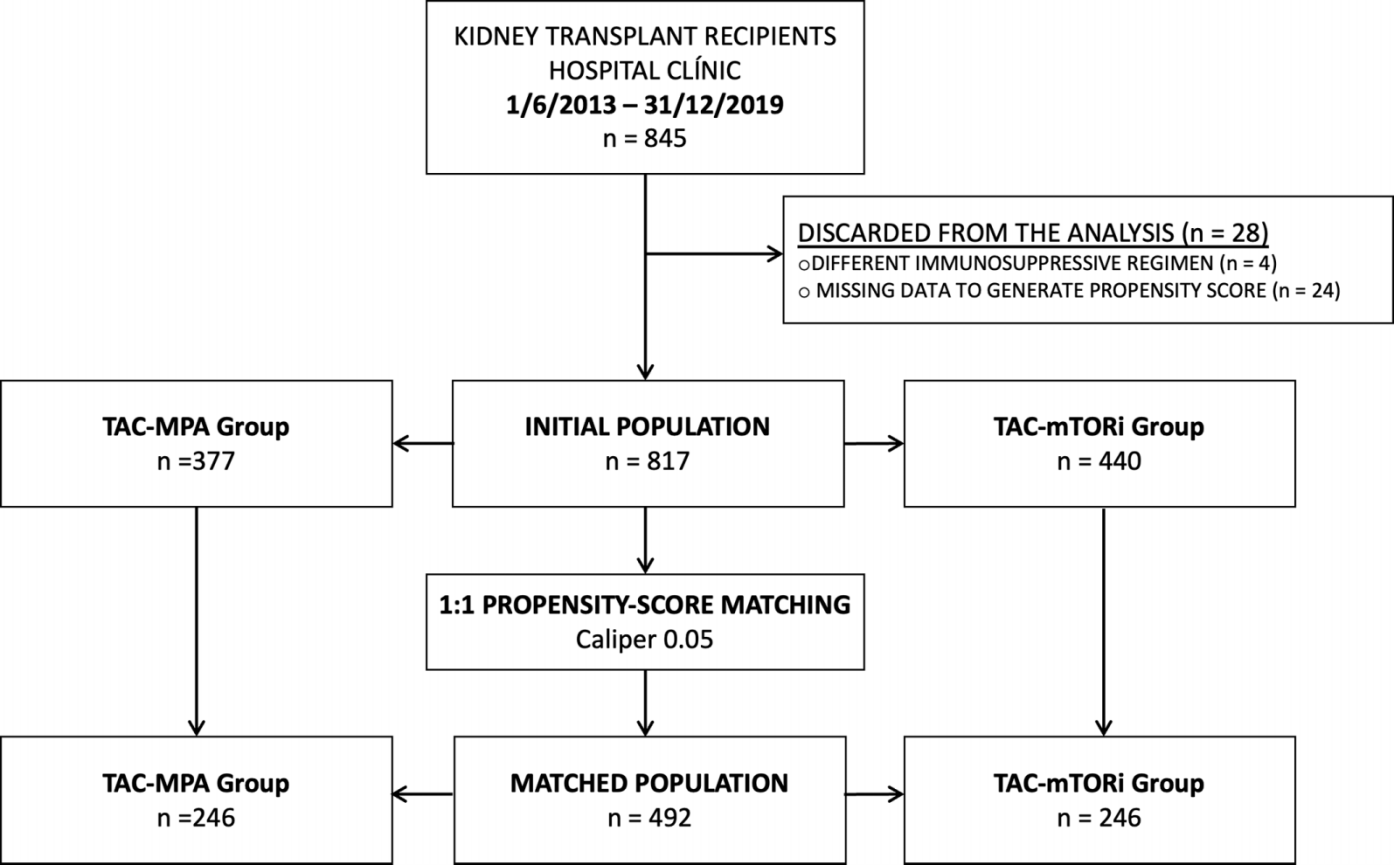
Flowchart of the included patients in the study period. TAC, tacrolimus; MPA, mycophenolic acid; TORi, and mTOR inhibitors.

All patients who received a solitary kidney transplant at Hospital Clínic of Barcelona between 1st June 2013, and 31^st^ December 2019, were initially screened for inclusion in this study (*n* = 845). Patients were excluded from the final analysis if they had insufficient data for propensity score calculation or received an immunosuppressive regimen other than tacrolimus plus mycophenolic acid or tacrolimus plus an mTOR inhibitor. From the final cohort, a 1:1 propensity score-matched analysis was performed as described in Sections “Statistical analysis” and “Baseline characteristics of the included population and propensity-score matching” (Fig. 1). The study was approved by the local Ethics Committee (HCB/2024/0390).

### Immunosuppressive treatment

According to our local protocol, induction therapy consists of either basiliximab (Simulect, Novartis, Basel, Switzerland; 20 mg on the day of transplantation and 20 mg on day 4) or anti-thymocyte globulin, administered as Thymoglobulin (1.25–1.5 mg/kg/day; Genzyme, Cambridge, UK) or Grafalon (2.5 mg/kg/day; Neovii, Gräfelfing, Germany) for five consecutive days, based on the patient’s immunological risk and donor type. HLA-identical recipients do not receive induction therapy. All patients receive an intraoperative pulse of methylprednisolone 500 mg, followed by 125 mg on postoperative day 1. This is followed by a tapering regimen starting at 0.5 mg/kg/day, with a reduction of 5 mg every 2 days until reaching 20 mg at 1 month post-transplant. The dose is then progressively tapered to 5 mg by the end of the third month. Tacrolimus, either Advagraf (Astellas, Leiden, The Netherlands) or Envarsus (Chiesi, Parma, Italy), is initiated before transplantation and combined with a third immunosuppressive agent: either an mTOR inhibitor [everolimus 1 mg twice daily (Certican, Novartis, Basel, Switzerland) or sirolimus 2 mg daily (Rapamune, Pfizer, New York, NY, USA)] or mycophenolate sodium (Myfortic, Novartis, Basel, Switzerland), both used as *de novo* agents. The mTORi dose was adjusted aiming at target through levels of 3–5 ng/ml. The choice of the third immunosuppressant depended on the clinical protocol active at the time of transplantation and the treating physician’s preference. Notably, in our center, mTORi are prescribed regardless of immunological risk. Contraindications for prescribing mTORi in our clinical protocol include obesity, focal segmental glomerulosclerosis as the underlying kidney disease, severe chronic obstructive pulmonary disease and a history of thrombotic microangiopathy.

### Cardiological work-up for transplantation clearance

According to our clinical protocol, all patients undergo a comprehensive medical history review and assessment of cardiovascular risk factors, along with an Electrocardiogram (ECG) and echocardiogram as part of the standard screening for transplant eligibility. Patients under 50 years of age, who are candidates for a first kidney transplant, have spent less than 1 year on dialysis and have no cardiovascular risk factors, are not routinely subjected to a stress-rest test. Patients aged 50 years or older may undergo a treadmill or stationary bicycle stress test if considered low risk—defined as being a first transplant candidate with no diabetes, smoking status and no prior cardiovascular events. High-risk patients, including those with diabetes, smoking status, a dialysis vintage >1 year, history of cardiovascular events, or a positive stress test, are referred for further evaluation with either SPECT or stress echocardiography. If these tests yield positive results, patients then undergo coronary angiography to assess coronary artery patency and determine whether revascularization (percutaneous or surgical) is indicated.

### Follow-up and study end-point

The primary outcome, MACE, was defined as the occurrence of non-fatal myocardial infarction, non-fatal stroke, or cardiovascular death. Secondary outcomes included each individual component of the MACE definition, as well as all-cause mortality. Patients were followed until the occurrence of the event, censoring at death, or until 31st December 2022, whichever came first.

### Statistical analysis

Continuous variables were described according to their distribution: variables with a normal distribution were presented as mean ± standard deviation, while those not following a normal distribution were reported as median and interquartile range. Categorical variables were summarized using absolute frequencies and percentages. A propensity score for receiving mTORi in combination with TAC was developed based on 18 baseline variables: donor and recipient age and sex, body mass index (BMI), diabetes mellitus, previous transplantation, etiology of chronic kidney disease, type of dialysis prior to transplantation, type of donor, calculated panel reactive antibodies (cPRA) at transplantation, HLA-A/B/DR mismatches, dialysis vintage, history of acute myocardial infarction (AMI), previous stroke, smoking status (active/former), coronary revascularization as part of the pre-transplant evaluation, and induction immunosuppressive therapy. Patients treated with TAC + mTORi and TAC + MPA were matched 1:1 using a caliper of 0.05 [12]. The adequacy of covariate balance following matching was evaluated using absolute standardized differences (ASD), with values <0.10 considered indicative of optimal balance. Inter-group comparisons were also assessed by means of Student’s *t*-test for normal variables or Mann–Whitney test for variables with non-parametric distribution, while differences for categorical variables were analyzed with Fisher’s exact test. The outcomes of interest were analyzed using Cox proportional hazards regression models, with results expressed as hazard ratios (HRs) and 95% confidence intervals (CIs). Continuous variables were dichotomized using the upper tertile, which was considered the risk-exposed category in Cox regression models. All statistical tests were two-tailed, and a *P*-value <0.05 was considered statistically significant. All analyses were performed using RStudio (Version 2024.04.2 + 764) with the packages *MatchIt* and *tableone*. Time-to-event survival curve were censored at death or at the last follow-up visit and were generated using GraphPad Prism v5, as well as Forest Plot (GraphPad Software, La Jolla, CA, USA).

## RESULTS

### Baseline characteristics of the included population and propensity-score matching

**Figure 2:**
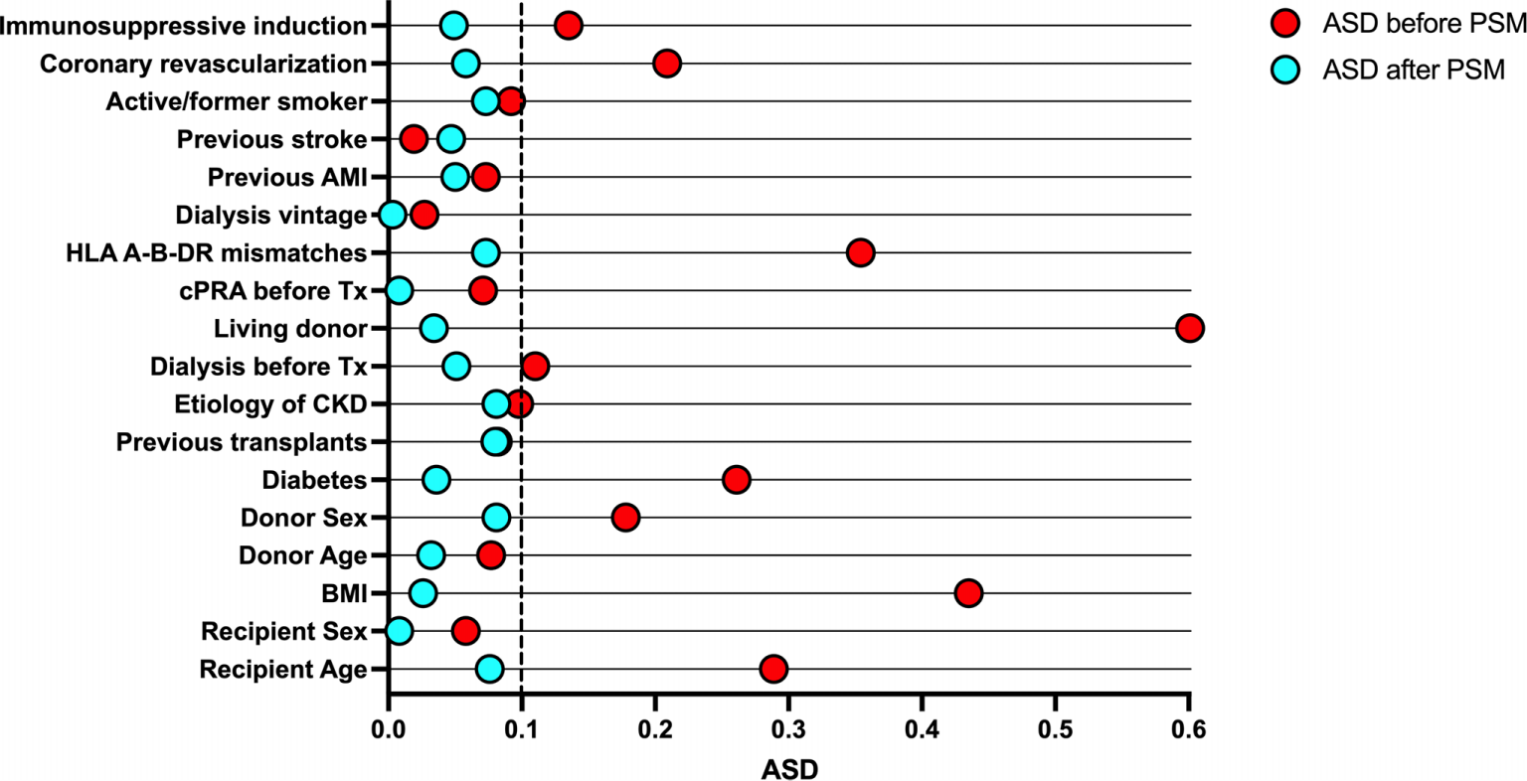
Dotchart of baseline imbalances’ ASD before and after PSM.

From the original cohort of 845 patients, 28 were excluded because of different immunosupressive regimens (*n* = 4) and insufficient data to generate the propensity score (*n* = 24) (Fig. 1). The remaining 817 patients demonstrated baseline imbalances that could potentially favor either the MPA or mTORi groups in terms of cardiovascular risk after transplantation (Supplementary Table S1). Specifically, the MPA group appeared advantaged by a younger age and a higher prevalence of living donors, while the mTORi group was favored by a lower BMI and a lower prevalence of diabetes. Following 1:1 propensity score matching (PSM) based on 18 baseline variables, a final cohort of 492 patients was obtained, showing adequate balance across all covariates, with all ASD <0.10 (Fig. 2). Importantly, no significant differences were observed in key cardiovascular risk factors, including age, sex, diabetes, BMI, dialysis vintage, smoking status, prior stroke or AMI, and coronary revascularization as a result of the pre-transplant work-up (Supplementary Table S1). Steroid was withdrawn in 10.6% of patients of the MPA group and 7.2% of the mTORi group (*P* = 0.269).

### Cardiovascular work-up before transplantation

In the propensity score-matched (PSM) population, 42.3% of patients were classified as low-risk and did not undergo any ischemia testing. A smaller proportion (11.7%) underwent only a stress test, either on a treadmill or stationary bicycle, while the relative majority (45.9%) underwent SPECT, stress echocardiography, or both. No significant differences were observed between the mTORi and MPA groups regarding the type of pre-transplant cardiovascular work-up (*P* = 0.360). A total of 62 patients (12.9%) eventually underwent coronary angiography as a result of the pre-transplant work-up. Among these, 23 patients (4.7%) required revascularization, performed either percutaneously (3.3%) or surgically (1.4%), before being definitively cleared for transplantation. There were no significant differences between the mTORi and MPA groups regarding the need for revascularization, whether assessed in the entire matched population or restricted to those who underwent coronary angiography (Table 2).

**Table 1:** Baseline: characteristics of the included population included in the PSM algorithm.

	TAC + MPA (*n* = 246)	TAC + mTORi (*n* = 246)	ASD
**Recipient age (years)**	56.6 ± 12.9	55.6 ± 13.3	0.076
**Recipient sex (%males)**	151/246 (61.4%)	150/246 (61.0%)	0.008
**Body mass index**	25.6 ± 4.5	25.5 ± 4.0	0.026
**Donor age (years)**	59.7 ± 12.9	59.3 ± 12.9	0.032
**Donor sex (%males)**	124/246 (50.4%)	114/246 (46.3%)	0.081
**Diabetes (%yes)**	66/246 (26.8%)	70/246 (28.5%)	0.036
**Previous Tx**			0.080
– 0	171/246 (69.5%)	180/246 (73.2%)	
– 1	56/246 (22.8%)	45/246 (18.3%)	
– ≥2	19/246 (7.7%	21/246 (8.5%)	
**Etiology of CKD**			0.081
– Unknown	59/246 (24.0%)	50/246 (20.3%)	
– Genetic	19/246 (7.7%)	47/246 (19.1%)	
– Immunologic	76/246 (30.9%)	50/246 (20.3%)	
– Diabetes	39/246 (15.9%)	46/246 (18.7%)	
– Hypertensive	32/246 (13.0%)	29/246 (11.8%)	
– Other	21/246 (8.5%)	24/246 (9.8%)	
**Dialysis before Tx (%)**			0.051
– No (pre-emptive)	51/246 (20.7%)	52/246 (21.1%)	
– Hemodialysis	165/246 (67.1%)	159/246 (64.6%)	
– Peritoneal dialysis	30/246 (12.2%)	35/246 (14.2%)	
**Living donor (%yes)**	86/246 (35.0%)	82/246 (33.3%)	0.034
**cPRA before Tx (%)**	0 [0–64]	0 [0–67.75]	0.008
**HLA A-B-DR mismatches (n)**	3.9 ± 1.6	4.0 ± 1.4	0.073
**Dialysis vintage (months)**	25 [7–60]	26 [5–59]	0.003
**Previous AMI (%yes)**	32/246 (13.0%)	28/246 (11.4%)	0.050
**Previous stroke (%yes)**	16/246 (6.5%)	19/246 (7.7%)	0.047
**Active/Former smoker (%yes)**	115/246 (46.7%)	124/246 (50.4%)	0.073
**Coronary revascularization after pre-Tx work-up (%yes)**	13/246 (5.3%)	10/246 (4.1%)	0.058
**Immunosuppressive induction (%)**			0.049
– No induction	15/246 (6.1%)	9/246 (3.7%)	
– Anti-thymocyte globulins	136/246 (55.3%)	142/246 (57.7%)	
– Anti-CD25	95/246 (38.6%)	95/246 (38.6%)	

Cases and controls from the original population (*n* = 841) have been matched 1:1 with a caliper set to ≤0.05. Adequate balance between the matched cases and controls was assessed by means of ASD and an ASD <0.10 was considered as optimal. BMI, body mass index; cPRA, calculated panel reactive antibodies; CKD, chronic kidney disease; TAC, tacrolimus; MPA, mycophenolate; mTORi, mTOR inhibitors; and Tx, transplantation.

**Table 2:** Description: of the cardiologic work-up for patients’ clearance before transplantation.

	TAC + MPA (*n* = 246)	TAC + mTORi (*n* = 246)	*P*-value
**Ischemia Study (%)**			0.360
– No	113/246 (45.9%)	95/246 (38.6%)	
– Stress test	24/246 (9.8%)	34/246 (13.8%)	
– SPECT	83/246 (33.7%)	102/246 (41.5%)	
– Stress echocardiography	23/246 (9.3%)	13/246 (5.3%)	
– All of the above	3/246 (1.2%)	2/246 (0.8%)	
**Clinical decision making after ischemia work-up before transplantation**			
– **Coronary angiography (%yes)**	36/246 (14.6%)	26/246 (10.6%)	
– **Revascularization—Total population (%yes)**			0.561
– No	233/246 (94.7%)	236/246 (95.95%)	
– Stent	8/246 (3.3%)	8/246 (3.3%)	
– Bypass	5/246 (2.0%)	2/246 (0.8%)	
– **Revascularization—Patients who underwent coronary angiography (%yes)**			1
– No	23/36 (63.9%)	16/26 (61.5%)	
– Stent	8/36 (22.2%)	8/26 (30.8%)	
– Bypass	5/36 (13.9%)	2/26 (7.7%)	

TAC, tacrolimus; MPA, mycophenolate; and mTORi, mTOR inhibitors.

### Immunosuppression management after transplantation

During the first year following KT, a total of 138 patients underwent changes in their immunosuppressive regimen, with a significantly higher proportion in the mTORi group (37.4%) compared to the MPA group (18.7%) (*P* < 0.001). The reasons for these changes are detailed in Supplementary Table S2. Interestingly, approximately one-third of immunosuppression changes in the mTORi group were attributed to either physician preference or graft-related issues, such as acute rejection, decline in renal function, or progressive fibrosis on renal biopsy. However, current evidence suggests that *de novo* use of mTORi in combination with CNI is not associated with an increased risk of these events.^6^ Therefore, the relatively high rate of changes in the mTORi group may reflect variability in physicians’ attitude towards their use. Regarding TAC levels, trough concentrations during the first year were consistently lower in the mTORi group compared to the MPA group at most time points. At 12 months post-transplant, the mean TAC trough level was 6.91 ± 2.64 ng/ml in the mTORi group vs. 7.80 ± 3.00 ng/ml in the MPA group (*P* = 0.001) (Table 3).

**Table 3:** TAC: and mTORi trough levels during the first year after kidney transplantation.

	TAC trough levels		
	MPA group	mTORi group	*P*-value
**Week 1**	9.40 ± 4.87	8.48 ± 4.30	0.014
**Week 2**	9.78 ± 5.38	8.27 ± 3.53	<0.001
**Month 1**	10.04 ± 3.44	9.05 ± 3.32	<0.001
**Month 2**	9.35 ± 3.30	8.73 ± 3.25	0.021
**Month 4**	8.33 ± 3.39	8.33 ± 3.54	0.490
**Month 6**	8.17 ± 3.52	7.32 ± 3.17	0.004
**Month 9**	8.08 ± 3.56	7.26 ± 2.60	0.004
**Month 12**	7.80 ± 3.00	6.91 ± 2.64	0.001

TAC, tacrolimus; MPA, mycophenolate; and mTORi, mTOR inhibitors.

### Baseline immunosuppression is not associated with post-transplant MACE

**Figure 3:**
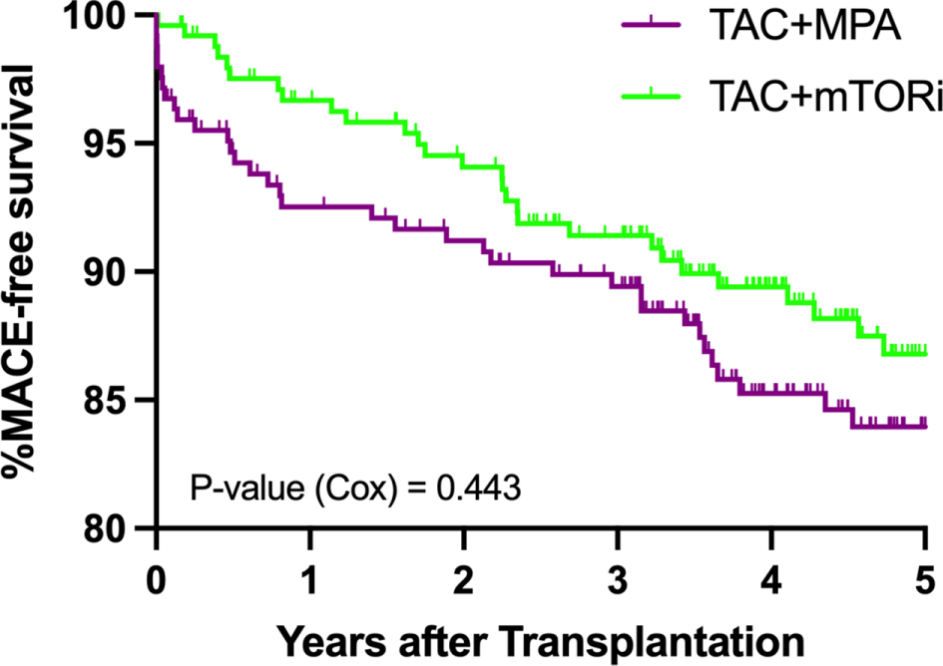
Kaplan–Meier estimates of 5-year rejection free survival according to treatment group (TAC + MPA vs. TAC + mTORi). TAC, tacrolimus; MPA, mycophenolic acid; and mTORi, mTOR inhibitor.

Patients were followed for a median of 4.81 ± 2.49 years. During this period, MACE occurred in 78 patients (15.9%), with no significant difference between the mTORi and MPA groups (HR 0.84[95% CI: 0.54–1.30], *P* = 0.443) (Fig. 3). Ischemic heart disease occurred in 42 patients (8.5%), presenting as unstable angina (3.3%) or AMI (5.3%). Among those with AMI, 73.1% had a Killip class I–II at presentation, while 26.9% presented with more severe heart failure (Killip class III–IV). Stroke occurred in 19 patients (3.9%), and cardiovascular-related death was recorded in 36 patients (7.3%). There were no significant differences between treatment groups for any of these cardiovascular outcomes (Table 4). All-cause mortality occurred in 106 patients (21.6%), again without a statistically significant difference between the mTORi and MPA groups (*P* = 0.241). In a sensitivity analysis restricted to patients who maintained their initial immunosuppressive regimen during the first year (*n* = 354), MACE occurred in 33 patients (16.5%) in the MPA group and 20 patients (13.0%) in the mTORi group. Cox regression analysis showed no significant difference (HR 0.72[95% CI: 0.41–1.25], *P* = 0.249). Further sensitivity analyses revealed no differences in outcomes between treatment groups among patients with diabetes (*n* = 136; HR 0.99[95% CI: 0.52–1.90], *P* = 0.993) or without diabetes (*n* = 356; HR 0.69[95% CI: 0.38–1.26], *P* = 0.228). Similarly, no differences were observed among patients who did not undergo ischemia testing as part of the pre-transplant work-up (HR 0.99[95% CI: 0.44–2.23], *P* = 0.998) and those who did undergo ischemia testing (HR 0.71[95% CI: 0.42–1.20], *P* = 0.203). Among patients with a history of stroke or AMI prior to transplantation (*n* = 86), post-transplant MACE occurred in 28 cases (32.6%), showing no significant differences between the groups (HR 0.74[95% CI: 0.35–1.57], *P* = 0.443). Assessment of 24-h proteinuria at 1 year after transplantation the per-protocol population showed no difference (MPA vs. mTORi 160[90–332] vs. 174 [94–448]mg, *P* = 0.400),

**Table 4:** Post-transplant: outcomes in the studied population.

	TAC + MPA (*n* = 246)	TAC + mTORi (*n* = 246)	HR [95%] C.I. mTORi vs. MPA	*P*-value
**MACE (%yes)**	43/246 (17.5%)	38/246 (15.4%)	0.84[0.54–1.30]	0.443
**Ischemic heart disease (%)**	22/246 (8.9%)	20/246 (8.1%)	0.87[0.47–1.60]	0.660
– Unstable angina	9/22 (40.9%)	7/20 (35.0%)	0.75[0.28–2.01]	0.569
– Acute myocardial infarction	13/22 (59.1%)	13/20 (65.0%)	0.96[0.44–2.07]	0.958
– Killip category				
– 1	7/13 (53.8%)	8/13 (61.5%)		
– 2	1/13 (7.7%)	3/13 (23.1%)		
– 3	4/13 (30.8%)	1/13 (7.7%)		
– 4	1/13 (7.7%)	1/13 (7.7%)		
**Stroke (%yes)**	8/246 (3.3%)	11/246 (4.5%)	1.30[0.52–3.23]	0.571
**Death for cardiovascular cause (%yes)**	21/246 (8.5%)	15/246 (6.5%)	0.72 [0.37–1.39]	0.335
**Death (any cause)**	58/246 (23.6%)	48/246 (19.5%)	0.79 [0.54–1.16]	0.241

MACE were defined as the occurrence of either ischemic heart disease (AMI or unstable angina), stroke or death from any cardiovascular cause. TAC, tacrolimus; MPA, mycophenolic acid; and mTORi, mTOR inhibitors.

### Pre-transplant risk factors and graft quality predict post-transplant MACE

**Figure 4:**
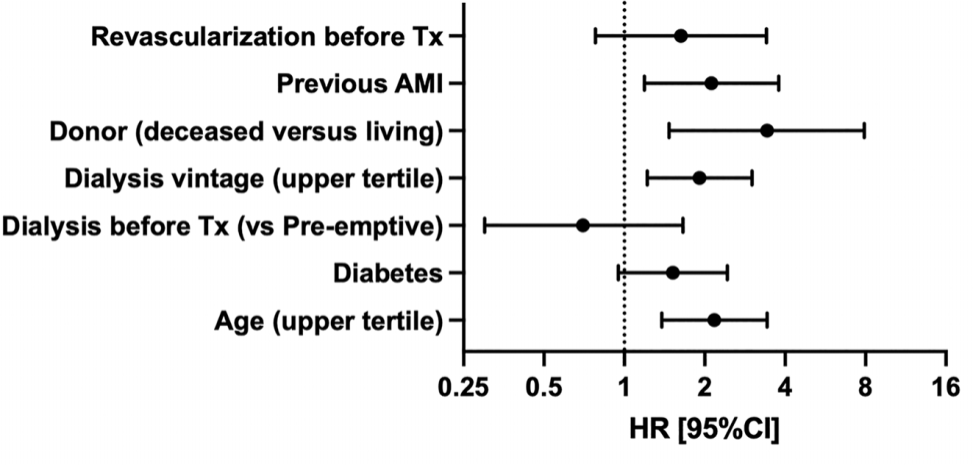
Forest plot of hazard ratios of risk factors for the development of MACE. Tx, transplant; AMI, acute myocardial infarction; and CI, confidence interval.

When analyzing all known risk factors for the development of MACE using univariable Cox regression, several baseline characteristics were significantly associated with the outcome. These included age in the upper tertile (HR 2.98[95% CI 1.92–4.61], *P* < 0.001), diabetes (HR 2.76[1.78–4.28], *P* < 0.001), dialysis before transplantation vs. pre-emptive transplantation (HR 2.40[1.20–4.80], *P* = 0.013), prolonged dialysis vintage (HR 2.42[1.57–3.75], *P* < 0.001), deceased donor vs. living donor (HR 4.93[2.53–9.60], *P* < 0.001), prior AMI (HR 3.39[2.09–5.49], *P* < 0.001), and coronary revascularization as part of the pre-transplant work-up (HR 4.30[2.33–7.95], *P* < 0.001) (Supplementary Table S3). In the multivariable model, the following factors remained independently associated with increased MACE risk: age in the upper tertile (HR 2.17[1.38–3.42], *P* < 0.001), prolonged dialysis vintage (HR 1.91[1.22–3.01], *P* = 0.005), deceased donor status (HR 3.42[1.47–7.92], *P* = 0.004), and prior AMI (HR 2.12[1.19–3.78], *P* = 0.011). Notably, diabetes showed a non-significant trend in the adjusted model (HR 1.52[0.95–2.43], *P* = 0.078) (Fig. 4).

## DISCUSSION

During the past years, the mTORi have been studied for their putative role in preventing or reducing atherosclerosis and cardiac hypertrophy. In fact, everolimus-coated stents are currently used in the field of interventional cardiology, since they proved to be superior to both bare metal and paclitaxel stents [13–15]. The mTOR signaling in cardiovascular physiology and disease is exerted through two multiprotein complexes, mTOR complex 1 (mTORC1) and mTOR complex 2 (mTORC2). Inhibition of mTORC1 is associated with reduced macrophage recruitment into the atherosclerotic plaque [16, 17]. mTORC1 is also necessary for cardiac adaptation to pressure overload and development of compensatory hypertrophy [16, 17]. Pharmacological mTORC1 inhibition and knock-out models proved to reduce heart hypertrophy, rebalance metabolic derangements, and minimize chronic myocardial infarction, resulting in overall less heart failure and extended mammalian life span [8]. In humans, observational studies have demonstrated up to 30% left ventricle mass reduction in patients who were switched from CNI to mTORi [8]. In experimental rat studies, sirolimus demonstrated overall better aortic contraction, endothelial-dependent and -independent relaxation, and reduced interleukin (IL)-1β and tumor necrosis factor (TNF)-α levels compared to cyclosporine, tacrolimus, and everolimus. On the other hand, everolimus proved better in terms of aortic endothelial compromise and smooth muscle function [9]. Moreover, mTORi might also indirectly improve atherosclerosis progression and heart failure development by allowing reduction of CNI trough levels that contribute to graft loss and hypertension [6], a finding also demonstrated in our actual data (Table 3).

However, all these putative cardioprotective effects must be weighed against the metabolic side effects of mTOR inhibitors, which include disturbances in glucose and lipid metabolism that may promote the development and progression of atherosclerotic plaques [7]. Unfortunately, few clinical studies have directly addressed this question, and most existing data are derived from registry-based analyses. A large retrospective study using Korea’s National Insurance Database (*n* = 12 000) found that MACE incidence after KT was associated solely with baseline comorbidities, with no apparent contribution from immunosuppressive regimens. Notably, this analysis focused on a low-risk cohort, excluding patients with a history of MACE prior to transplantation [2]. More recently, another study from the Korean registry reported that sirolimus use between 2010 and 2021 was not associated with a reduced incidence of MACE [11]. However, in that study, sirolimus was administered in combination with either mycophenolate or tacrolimus, complicating interpretation. Given that the combination of sirolimus and mycophenolate has been associated with increased toxicity and a higher risk of acute rejection, as demonstrated in the SYMPHONY trial [18], definitive conclusions regarding the cardiovascular effects of sirolimus in this setting remain difficult to establish.

Here, we addressed this critical question using a deeply phenotyped cohort and a PSM algorithm to balance 18 key covariates potentially associated—either directly or indirectly—with the outcome. Notably, our dataset included detailed information on pre-transplant cardiovascular work-up, previous cardiovascular events, and the need for revascularization as part of pre-transplant evaluation, providing a robust and clinically relevant snapshot derived from real-world practice (Table 2). Our findings demonstrated no significant difference in MACE incidence between the two immunosuppressive strategies—MPA or mTORi—both used in combination with tacrolimus (Table 4). Subgroup analyses confirmed the consistency of this result across patients stratified by diabetes, history of CVD, pre-transplant cardiological evaluation, and post-transplant immunosuppression modifications. When analyzing all putative factors associated with MACE development, we found that MACE occurrence was primarily driven by pre-transplant, non-modifiable risk factors—namely, older age, prolonged dialysis vintage, and a history of myocardial infarction—as well as graft quality, with living donor transplants being naturally favored. Curiously, pre-transplant diabetes did not reach statistical significance at multivariable analysis (Fig. 4), either suggesting that its role is partially offset by stronger risk factors or the sample size was not adequately powered to show this interaction. Importantly, we did not observe increased proteinuria in the mTORi patients’ group, a common side effect usually found in early trials, in which mTORi trough levels were considerably higher than in current clinical practice [19]. Our results are in line with those of the recently published OPTIMIZE trial that use an optimized mTORi trough levels target (3–6 ng/ml) [20], highlighting that this side effect may be dose-related. Taken together, our results suggest that the potential cardiovascular benefits of mTOR inhibitors demonstrated in experimental models, such as regression of left ventricular hypertrophy and attenuation of atherosclerosis [8–10], may be counterbalanced by their adverse effects on glucose and lipid metabolism. As a result, the overall impact of mTOR inhibitors on MACE risk appears to be neutral when compared to MPA in the clinical setting.

Our findings should be interpreted in light of the study’s limitations, most notably its retrospective design and the high rate of immunosuppression changes observed in the TAC + mTORi group. This phenomenon has been previously reported [6], and may be partially attributed to physician preference, as reflected both in our cohort and in a post hoc analysis of the TRANSFORM trial [21]. Another limitation is the absence of pre- and post-transplant echocardiographic data on left ventricular remodeling, which limits interpretation of the putative beneficial cardiovascular effects associated with mTOR inhibitor use [8]. External validity should be considered in light of center-specific practices. In particular, indications for mTOR inhibitor use and cardiovascular screening protocols vary across transplant centers, which may influence absolute event rates. Therefore, our findings are most directly applicable to centers adopting a de novo tacrolimus–mTOR inhibitor strategy and standardized cardiovascular assessment, while extrapolation to settings with different immunosuppressive or cardiovascular work-up practices should be made with caution. Despite these limitations, a key strength of our study lies in its comprehensive assessment of pre-transplant variables potentially influencing MACE, as well as its extended follow-up period. Together, these features provide valuable insights into the long-term cardiovascular impact of immunosuppressive strategies in a real-world clinical setting.

In conclusion, our study found no association between baseline immunosuppressive regimen and the development of MACE after KT. Instead, MACE incidence appeared to be primarily driven by classical, non-modifiable risk factors such as age, dialysis vintage, prior myocardial infarction, and graft quality. Further studies should aim to explore new approaches to tackle cardiovascular risk in kidney transplant recipients, with the final aim to prevent MACE after transplantation.

## Supplementary Material

sfag101_Supplemental_File

## Data Availability

De-identified data used in the current analysis will be made available to researchers after approval of a proposal for its use, according to the rules of the local Ethical Committee.

## References

[1] Anderson B, Qasim M, Evison F et al. A population cohort analysis of English transplant centers indicates major adverse cardiovascular events after kidney transplantation. Kidney Int. 2022;102:876–84. 10.1016/j.kint.2022.05.017 35716956

[2] Kim JE, Park J, Park S et al. De novo major cardiovascular events in kidney transplant recipients: a comparative matched cohort study. Nephrol Dial Transplant. 2023;38:499–506. 10.1093/ndt/gfac144 35396847

[3] Kovesdy CP. Epidemiology of chronic kidney disease: an update 2022. Kidney Int Suppl. 2022;12:7–11. 10.1016/j.kisu.2021.11.003 PMC9073222 35529086

[4] Birdwell KA, Park M. Post-transplant cardiovascular disease. Clin J Am Soc Nephrol. 2021;16:1878–89. 10.2215/CJN.00520121 34556500 PMC8729500

[5] Cohen E, Korah M, Callender G et al. Metabolic disorders with kidney transplant. Clin J Am Soc Nephrol. 2020;15:732–42. 10.2215/CJN.09310819 32284323 PMC7269213

[6] Pascual J, Berger SP, Witzke O et al. Everolimus with reduced calcineurin inhibitor exposure in renal transplantation. J Am Soc Nephrol. 2018;29:1979–91. 10.1681/ASN.2018010009 29752413 PMC6050928

[7] Cuadrado-Payán E, Diekmann F, Cucchiari D. Medical aspects of mTOR inhibition in kidney transplantation. Int J Mol Sci. 2022;23:7707. 10.3390/ijms23147707 35887051 PMC9322634

[8] Sciarretta S, Volpe M, Sadoshima J. Mammalian target of rapamycin signaling in cardiac physiology and disease. Circ Res. 2014;114:549–64. 10.1161/CIRCRESAHA.114.302022 24481845 PMC3995130

[9] Shing CM, Fassett RG, Brown L et al. The effects of immunosuppressants on vascular function, systemic oxidative stress and inflammation in rats. Transpl Int. 2012;25:337–46. 10.1111/j.1432-2277.2011.01420.x 22239125

[10] Kurdi A, De Meyer GRY, Martinet W. Potential therapeutic effects of mTOR inhibition in atherosclerosis. Br J Clin Pharma. 2016;82:1267–79. 10.1111/bcp.12820 PMC5061792 26551391

[11] Park J, Choi W, Hwang J et al. Impact of sirolimus on long-term adverse cardiovascular outcomes in kidney transplant recipients: a nationwide cohort study. Eur J Clin Invest. 2025;55:e70027. 10.1111/eci.70027 40105194 PMC12169092

[12] Austin PC. Balance diagnostics for comparing the distribution of baseline covariates between treatment groups in propensity-score matched samples. Stat Med. 2009;28:3083–107. 10.1002/sim.3697 19757444 PMC3472075

[13] Stone GW, Kedhi E, Kereiakes DJ et al. Differential clinical responses to everolimus-eluting and Paclitaxel-eluting coronary stents in patients with and without diabetes mellitus. Circulation. 2011;124:893–900. 10.1161/CIRCULATIONAHA.111.031070 21824922

[14] Rodriguez AE, Palacios I, Rodriguez-Granillo AM et al. Comparison of cost-effectiveness of oral rapamycin plus bare-metal stents versus first generation of drug-eluting stents (from the randomized oral rapamycin in argentina [ORAR] 3 trial). Am J Cardiol. 2014;113:815–21. 10.1016/j.amjcard.2013.11.033 24528614

[15] Wessely R, Kastrati A, Mehilli J et al. Randomized trial of rapamycin- and paclitaxel-eluting stents with identical biodegradable polymeric coating and design. Eur Heart J. 2007;28:2720–5. 10.1093/eurheartj/ehm425 17921531

[16] Wang S, Amato KR, Song W et al. Regulation of endothelial cell proliferation and vascular assembly through distinct mTORC2 signaling pathways. Mol Cell Biol. 2015;35 :1299–313. 10.1128/MCB.00306-14 25582201 PMC4355541

[17] Laplante M, Sabatini DM. mTOR signaling at a glance. J Cell Sci. 2009;122(Pt 20):3589–94. 10.1242/jcs.051011 19812304 PMC2758797

[18] Ekberg H, Tedesco-Silva H, Demirbas A et al. Reduced exposure to calcineurin inhibitors in renal transplantation. N Engl J Med. 2007;357:2562–75. 10.1056/NEJMoa067411 18094377

[19] Diekmann F, Andrés A, Oppenheimer F. mTOR inhibitor-associated proteinuria in kidney transplant recipients. Transplant Rev (Orlando). 2012; 26:27–29. 22. 10.1016/j.trre.2011.10.003 22137729

[20] Sanders JF, de Boer SE, Jonker J et al. Immunosuppression in older kidney transplant recipients: a randomized controlled trial. J Am Soc Nephrol. 2025. 10.1681/ASN.0000000924 PMC13065175 41201871

[21] Tedesco-Silva H, Pascual J, Viklicky O et al. Safety of everolimus with reduced calcineurin inhibitor exposure in de novo kidney transplants: an analysis from the randomized TRANSFORM study. Transplantation. 2019;103:1953–63. 10.1097/TP.0000000000002626 30801548

