# Supplementary material for: Neutral impact of mTOR inhibitors on cardiovascular outcomes after kidney transplantation
Source: Clin Kidney J. 2026 Mar 25;19(4):sfag101. doi: 10.1093/ckj/sfag101 (PMC13100659; doi:10.1093/ckj/sfag101)
Supplement: sfag101_Supplemental_File [file sfag101_supplemental_file.docx]

|  | **TAC+MPA**  **(n=377)** | **TAC+mTORi**  **(n=440)** | **P Value** |
| --- | --- | --- | --- |
| **Recipient Age (years)** | 54.3 ± 13.9 | 58.2 ± 13.0 | <0.001 |
| **Recipient Sex (%males)** | 236/377 (62.6%) | 263/440 (59.8%) | 0.451 |
| **BMI** | 26.6 ± 5.0 | 24.7 ± 3.8 | <0.001 |
| **Donor age (years)** | 59.0 ± 12.7 | 60.0 ± 13.7 | 0.273 |
| **Donor sex (%males)** | 173/377 (45.9%) | 241/440 (54.8%) | 0.014 |
| **Diabetes (%yes)** | 121/377 (32.1%) | 91/440 (20.7%) | <0.001 |
| **Previous Tx (%)** |  |  | 0.228 |
| - 0 | 263/377 (69.8%) | 330/440 (75.0%) |  |
| - 1 | 77/377 (20.4%) | 77/440 (17.5%) |  |
| - ≥2 | 37/377 (9.8%) | 33/440 (7.5%) |  |
| **Etiology of CKD** |  |  | <0.001 |
| - Unknown | 87/377 (23.1%) | 104/440 (23.6%) |  |
| - Genetic | 30/377 (8.0%) | 88/440 (20.0%) |  |
| - Immunologic | 109/377 (28.9%) | 91/440 (20.7%) |  |
| - Diabetes | 69/377 (18.3%) | 61/440 (13.9%) |  |
| - Hypertensive | 38/377 (10.1%) | 55/440 (12.5%) |  |
| - Other | 44/377 (11.7%) | 41/440 (9.3%) |  |
| **Dialysis before Tx (%)** |  |  | 0.084 |
| No (pre-emptive) | 81/377 (21.5%) | 68/440 (15.5%) |  |
| Hemodialysis | 247/377 (65.5%) | 311/440 (70.7%) |  |
| Peritoneal dialysis | 49/377 (13.0%) | 61/440 (13.9%) |  |
| **Living donor (%yes)** | 198/377 (52.5%) | 351/440 (79.8%) | <0.001 |
| **cPRA before Tx (%)** | 0.0 [0.0–64.0] | 0.0 [0.0–54.8] | 0.116 |
| **HLA A-B-DR mismatches (n)** | 3.7 ± 1.6 | 4.2 ± 1.3 | <0.001 |
| **Dialysis vintage (months)** | 22.0 [4.0–59.0] | 29.0 [10.0–53.0] | 0.056 |
| **Previous AMI (%yes)** | 52/377 (13.8%) | 50/440 (11.4%) | 0.347 |
| **Previous stroke (%yes)** | 24/377 (6.4%) | 26/440 (5.9%) | 0.900 |
| **Active/Former smoker (%yes)** | 187/377 (49.6%) | 198/440 (45.0%) | 0.214 |
| **Coronary revascularization after pre-Tx work-up (%yes)** | 26/377 (6.9%) | 11/440 (2.5%) | 0.004 |
| **Immunosuppressive induction (%)** |  |  | 0.079 |
| No induction | 19/377 (5.0%) | 17/440 (3.9%) |  |
| Anti-thymocyte globulins | 203/377 (53.8%) | 271/440 (61.6%) |  |
| Anti-CD25 | 155/377 (41.1%) | 152/440 (34.5%) |  |

**Table S1** – Baseline characteristics of the initial population before the generation of the Propensity score matching algorithm. BMI, body mass index; cPRA, calculated panel reactive antibodies; CKD, chornic kidney disease; TAC, tacrolimus; MPA, mycophenolate; mTORi, mTOR inhibitors; Tx, transplantation.

|  | **TAC+MPA**  (n=246) | **TAC+mTORi**  (n=246) |
| --- | --- | --- |
| **Drug-related side-effects** | 15/246 (6.1%) | 67/246 (27.2%) |
| - Mucocutaneous alterations | 0/246 | 1/246 (0.4%) |
| - Bone marrow suppression | 3/246 (1.2%) | 1/246 (0.4%) |
| - Edemas | 0/246 | 13/246 (5.3%) |
| - Liver toxicity | 1/246 (0.4%) | 1/246 (0.4%) |
| - Lymphocele | 0/246 | 15/246 (6.1%) |
| - Surgical scar complications | 1/246 (0.4%) | 18/246 (7.3%) |
| - Pneumonitis | 0/246 | 6/246 (2.4%) |
| - Myositis | 0/246 | 1/246 (0.4%) |
| - Neurotoxicity | 2/246 (0.8%) | 1/246 (0.4%) |
| - Metabolic syndrome | 1/246 (0.4%) | 6/246 (2.4%) |
| - GI symptoms | 6/246 (2.4%) | 0/246 |
| - Serositis | 1/246 (0.4%) | 0/246 |
| **Clinical decision** | 14/246 (5.7%) | 30/246 (12.2%) |
| - Physician’s preference | 13/246 (5.3%) | 28/246 (11.4%) |
| - Pregnancy | 1/246 (0.4%) | 0/246 |
| - Planned surgical intervention | 0/246 | 2/246 (0.8%) |
| **Graft-related issues** | 2/246 (0.8%) | 17/246 (6.9%) |
| - Thrombotic microangiopathy | 1/246 (0.4%) | 4/246 (1.6%) |
| - Kidney biopsy alterations | 0/246 | 3/246 (1.2%) |
| - Proteinuria | 0/246 | 6/246 (2.4%) |
| - Renal function worsening | 0/246 | 5/246 (2.0%) |
| - Rejection | 1/246 (0.4%) | 3/246 (1.2%) |
| **Over-immunosuppression** | 30/246 (12.2%) | 8/246 (3.3%) |
| - CMV | 11/246 (5.3%) | 0/246 |
| - BK | 5/246 (2.0%) | 0/246 |
| - Other infecions | 5/246 (2.0%) | 7/246 (2.8%) |
| - Neoplasia | 9/246 (3.7%) | 1/246 (0.4%) |

**Table S2**- List of causes for immunosuppressive change during the first year after kidney transplantation in patients’ groups. TAC, tacrolimus; MPA, mycophenolic acid; mTORi, mTOR inhibitors.

|  | **Univariable** | | **Multivariable** | |
| --- | --- | --- | --- | --- |
|  | **HR [95% CI]** | **P-value** | **HR [95% CI]** | **P-value** |
| Age (upper tertile) | 2.98 [1.92–4.61] | **< 0.001** | 2.17 [1.38–3.42] | **< 0.001** |
| Sex (male) | 0.95 [0.61–1.49] | 0.829 |  |  |
| BMI (upper tertile) | 1.17 [0.74–1.84] | 0.500 |  |  |
| Diabetes | 2.76 [1.78–4.28] | **< 0.001** | 1.52 [0.95–2.43] | 0.078 |
| Smoking status | 1.26 [0.81–1.95] | 0.305 |  |  |
| Previous AMI | 3.39 [2.09–5.49] | **< 0.001** | 2.12 [1.19–3.78] | **0.011** |
| Previous stroke | 1.57 [0.75–3.25] | 0.229 |  |  |
| Dialysis vintage (upper tertile) | 2.42 [1.57–3.75] | **< 0.001** | 1.91 [1.22–3.01] | **0.005** |
| Previous Tx | 1.06 [0.65–1.72] | 0.808 |  |  |
| Dialysis before Tx (vs Pre-emptive) | 2.4 [1.2–4.8] | **0.013** | 0.7 [0.3–1.66] | 0.421 |
| Revascularization before Tx | 4.3 [2.33–7.95] | **< 0.001** | 1.63 [0.78–3.41] | 0.194 |
| Donor (deceased versus living) | 4.93 [2.53–9.6] | **< 0.001** | 3.42 [1.47–7.92] | **0.004** |
| Immunosuppression (mTOR vs MPA) | 0.84 [0.54–1.30] | 0.443 |  |  |

**Table S3** – Univariable and multivariable Cox regression analyses showing hazard ratios (HR) and 95% confidence intervals [CI] for the development of MACE based on common baseline risk factors. Continuous variables were categorized using the upper tertile as the cutoff. The thresholds used were: age > 63 years, BMI > 27.4 kg/m², and dialysis vintage > 42.3 months (~ 3.5 years).
